# High-throughput analysis of anti-poliovirus neutralization antibody titre in human serum by the pseudovirus neutralization test

**DOI:** 10.1038/s41598-022-20544-6

**Published:** 2022-09-27

**Authors:** Minetaro Arita, Masae Iwai-Itamochi

**Affiliations:** 1 Department of Virology II, National Institute of Infectious Diseases, 4-7-1 Gakuen, Musashimurayama-shi, Tokyo, 208-0011 Japan; 2 Department of Virology, Toyama Institute of Health, 17-1 Nakataikoyama, Imizu-shi, Toyama, 939-0363 Japan

**Keywords:** Policy and public health in microbiology, Inactivated vaccines, Clinical microbiology, Live attenuated vaccines, Microbiology, Infectious-disease diagnostics

## Abstract

To monitor vulnerability of countries to poliovirus (PV) outbreaks, serosurveillance of anti-PV neutralization antibody is conducted by conventional PV neutralization test (cPNT), which uses live PV strains. We previously developed a pseudovirus PV neutralization test (pPNT) as an alternative to cPNT, which uses PV pseudovirus that expresses luciferase as a reporter in the infection without producing infectious PV. In the present study, we established a high-throughput pPNT (HTpPNT) for a large-scale serosurveillance. The HTpPNT system was evaluated with 600 human serum samples obtained from a broad range of age groups of healthy volunteers (ages of 0–89 years). HTpPNT showed high correlation with cPNT (*R*^2^ for anti-type 1, 2, and 3 PV neutralization antibody titres are 0.90, 0.84, and 0.90, respectively). By using HTpPNT, we analyzed relative neutralizing antibody titre of the sera against a type 1 PV wild-type strain (Mahoney strain) to that against the type 1 Sabin strain. As a result, a correlation between the age (≥ 60 years) and the relative neutralizing antibody titre was observed (n = 15–16, *P* = 0.0000023–0.041), while the types of PV vaccine (i.e., oral PV vaccine and Sabin strain-based IPV) had no effect. HTpPNT would serve as a useful alternative to cPNT in a large-scale serosurveillance.

## Introduction

Poliovirus (PV) is a small non-enveloped virus with a positive-sense single-stranded RNA genome of approximately 7500 nt and belongs to the genus *Enterovirus*, the family *Picornaviridae*. PV is the causative agent of poliomyelitis, and is the target of the global eradication led by the World Health Organization (WHO) since 1988. Through the global vaccination program with live oral PV vaccine (OPV) and/or inactivated PV vaccine (IPV), as of 2022, only Pakistan and Afganistan remain as the endemic countries of wild PV. However, imported cases are still detected, such as the recent reports from Malawi and Mozambique. In Japan, Sabin strain-based IPV (sIPV) has been introduced in routine immunization since 2012 followed by the concomitant elimination of the vaccine-associated paralytic poliomyelitis cases^1^.

To monitor potential vulnerability to a poliomyelitis outbreak in Japan, annual national serosurveillance has been implemented in prefectural public health institutes since 1962, and approximately 1100–1800 individuals in 6–8 prefectures in a wide range of age groups (ages of 0–> 60) have been tested every year^2^. Until 1983, the serosurveillance was performed by using wild-type PV strains as the challenge viruses, which were then replaced with attenuated vaccine strains (Sabin strains). In 2015, the WHO published the third edition of the WHO Global Action Plan to minimize poliovirus facility-associated risk after type-specific eradication of wild polioviruses and sequential cessation of oral polio vaccine use (GAP III)^3^, which was recently revised as GAP IV^4^. The GAP III/ IV restricts the use of type 2 PV strains, including the Sabin 2 strain that causes the vast majority of circulating vaccine-derived PV (cVDPV) cases^5^, resulting in cessation of conventional PV neutralization test (cPNT) with the type 2 strains in the prefectural public health institutes in Japan; therefore, since 2017, the test has begun to be conducted only in the National Institute of Infectious Diseases. Subsequently, commercial services for the PV neutralization test in Japan have closed since 2019.

Under these circumstances, pseudovirus PV neutralization test (pPNT) has been established as an alternative to cPNT^6–10^. pPNT utilizes PV pseudovirus (PV_pv_) that possesses a luciferase-encoding PV replicon in the capsid and shows single cycle infection in susceptible cells (adsorption/uncoating/replication) without producing an infectious virus^11^. One of the major advantages of pPNT is the safety, which is free from infectious PV and allow the test to be performed in biosafety level (BSL) 2 laboratories. Neutralization tests with pseudotyped viruses have also been developed for other viruses, especially for those required to be handled in high BSL laboratories (e.g. Ebola virus, SARS-CoV, SARS-CoV-2, lyssaviruses, Nipah virus, highly pathogenic avian influenza A viruses)^12–18^.

Despite the feasibility of pPNT, a high-throughput system, which could be utilized in serosurveillance has yet to be established. In the present study, we automated most steps in pPNT and established the high-throughput system (HTpPNT); enable to test approximately 300 serum samples at a time by one to two persons. By using the HTpPNT, we analyzed the difference of neutralization antibody titres against a type 1 wild-type strain (Mahoney strain) and a vaccine strain (Sabin 1 strain) with human serum samples (a total of 731 samples) before and after the introduction of sIPV in routine immunization program.

## Results and discussion

### Automation of pPNT

**Figure 1:**
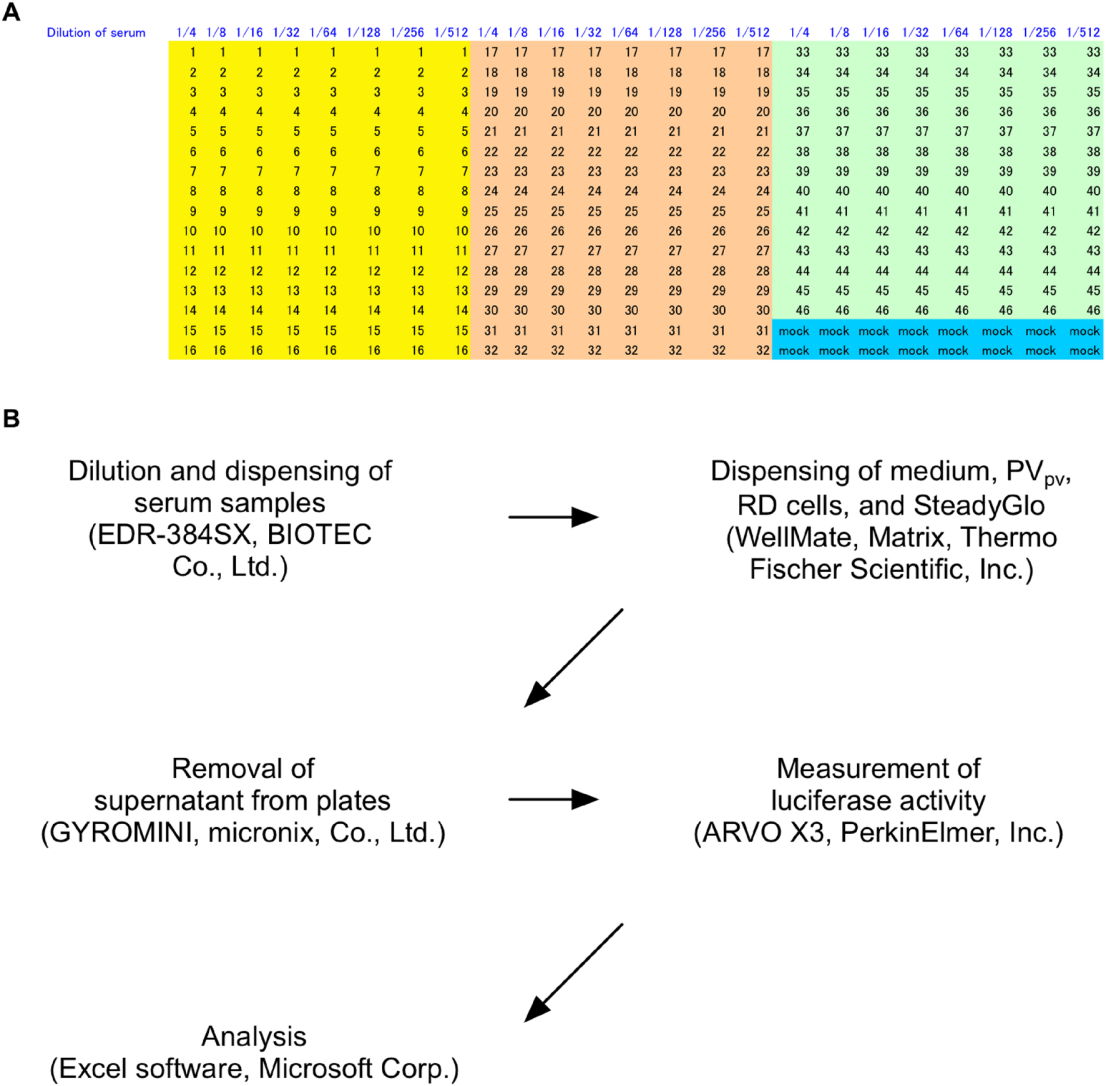
Automation of pPNT. (**A**) Plate layout of pPNT. Maximum 46 samples (including at least one sample of standard antisera in the test) were able to be tested per plate. (**B**) Equipment and software used for HTpPNT.

In the previous study, we established pPNT by manual handling^6,9^. To enable a large-scale pPNT which can be used in serosurveillance, we attempted to automate pPNT in the steps of serum dilution, plate copying, and removal of supernatant from the plates; an automatic dispenser/diluter/plate-copying machine (EDR-384SX work station) and a plate centrifuge for removal of supernatant (GYROMINI) were introduced (Fig. 1). Other equipment (dispenser and luminometer) were already employed in the previous systems^6,9^. The most difficult step for the automation was diluting human serum on a 384-well plate system, because serum shows a high resistance to be mixed due to a high concentration of high-molecular weight solutes including albumin (total protein concentration of about 6.5–8.0%)^19^. Human serum samples were mixed with 1% FCS-DMEM (fourfold dilution of the serum) by manual pipetting 10 times, and then subjected to the automatic diluter with mixing rate of 40 μL/s. Condition of the removal of supernatant by the plate centrifuge was optimized to recapitulate that by the manual decanting (1000 rpm for 1 s). These automations allowed testing of approximately 300 serum samples at a time by one to two persons.

### Evaluation of high-throughput pPNT (HTpPNT)

**Figure 2:**
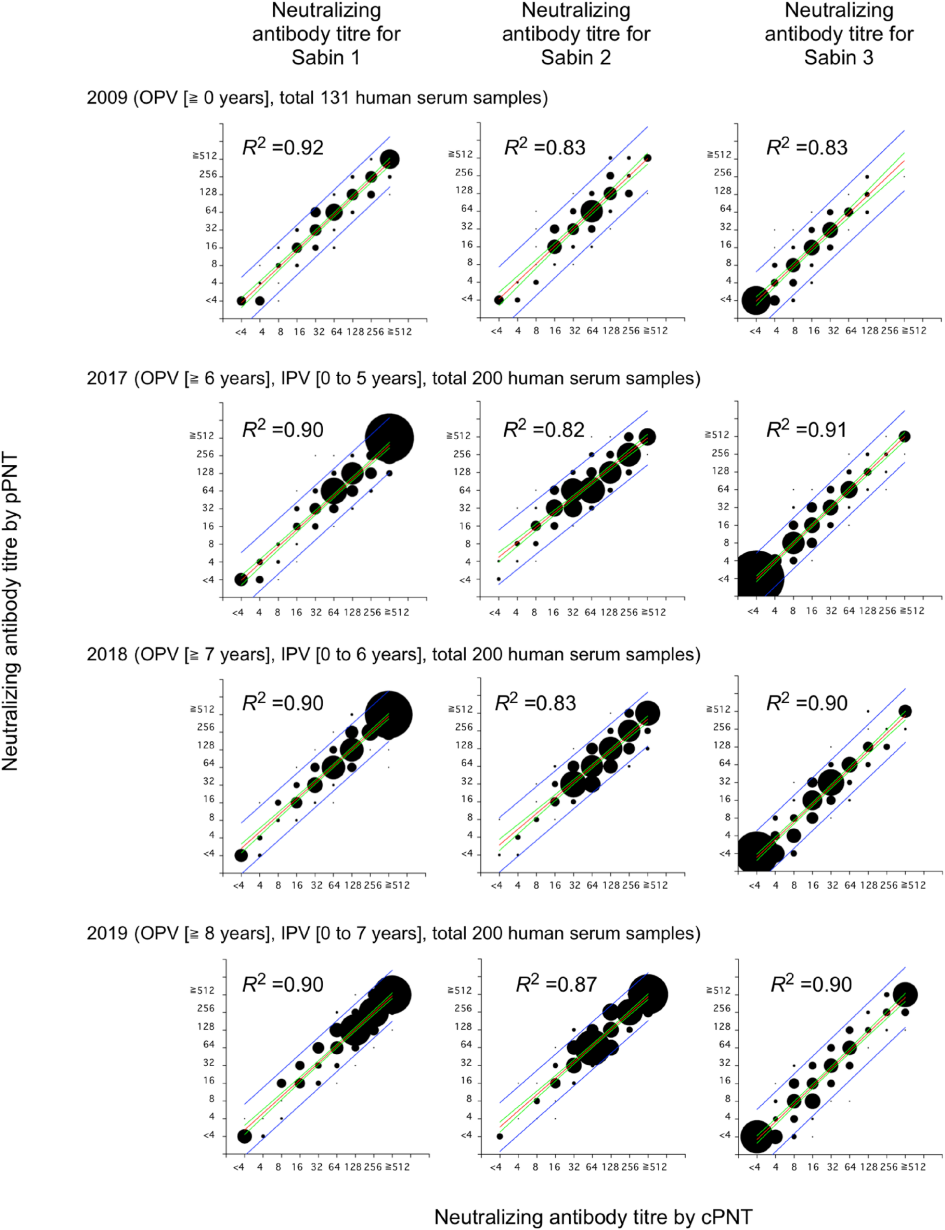
Comparison of results of cPNT and HTpPNT. Scatter plot of neutralizing antibody titres against PV by cPNT and HTpPNT. The size of the circles is proportional to the number of serum samples with the indicated titres. The coefficient of determination (*R*^2^), regression line (red), 95% confidence interval (green), and 95% prediction interval (blue) are shown.

**Figure 3:**
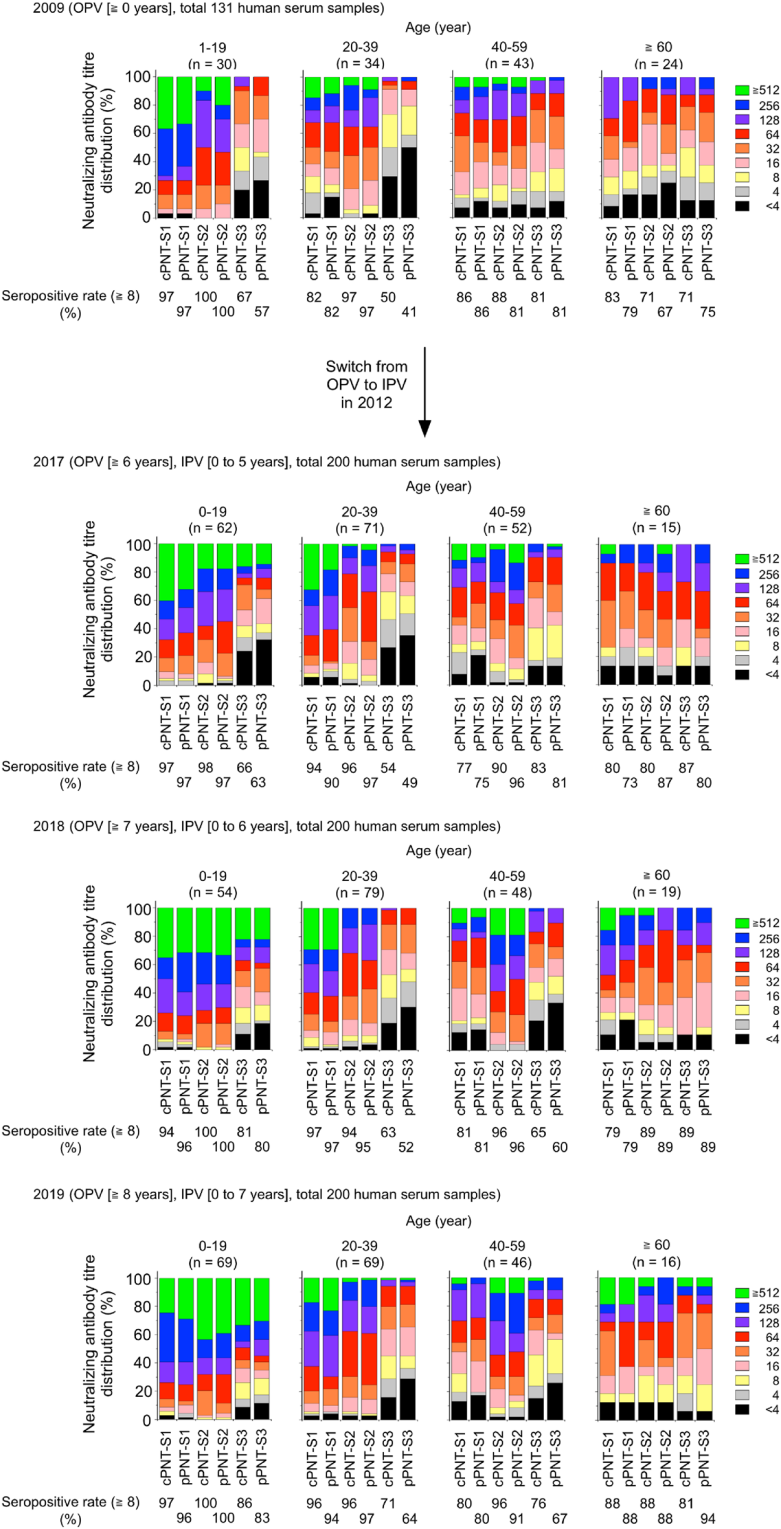
Transition of anti-PV neutralization antibody titre before and after the switch from OPV to sIPV in Japan. Distributions of neutralizing antibody titres against PV obtained by cPNT and HTpPNT in different age groups before and after the switch from OPV to sIPV in 2012 in routine immunization in Japan are shown.

To evaluate the utility of HTpPNT in serosurveillance, we compared the results of HTpPNT and those of cPNT by testing 600 human serum samples collected in 2017, 2018 and 2019 (Fig. 2, Supplementary Data 1, 2, 3 and 4). The results obtained by these tests showed high correlations, as previously observed for manual-handling pPNT (coefficient of determination *R*^2^ was 0.82–0.91)^9^. The high correlations were observed in the all age groups (Fig. 3, Supplementary Data 5, 6, 7 and 8). Marked increase was observed in the titre against the type 3 among the young age group (aged 0–19 years) after 2017 compared to that in 2009 due to the switch from OPV to sIPV in routine immunization program in 2012. According to the National serosurveillance, anti-PV neutralizing antibody titre is maintained at least until school-entry age in Japan (https://www.niid.go.jp/niid/ja/y-graphs/9664-polio-yosoku-serum2020.html), as well as the population vaccinated with cIPV (reviewed in^20^). Meanwhile, waning of the immunity in children/adolescents (mostly cIPV vaccinees, aged 1–14 years) has been observed possibly due to intrinsic nature of IPV without booster^21^. Therefore, further monitoring of the immunity induced by sIPV is essential to confirm the effectiveness.

### Analysis on difference of the neutralizing antibody titre against type 1 WT strain (Mahoney strain) and Sabin 1 strain

**Figure 4:**
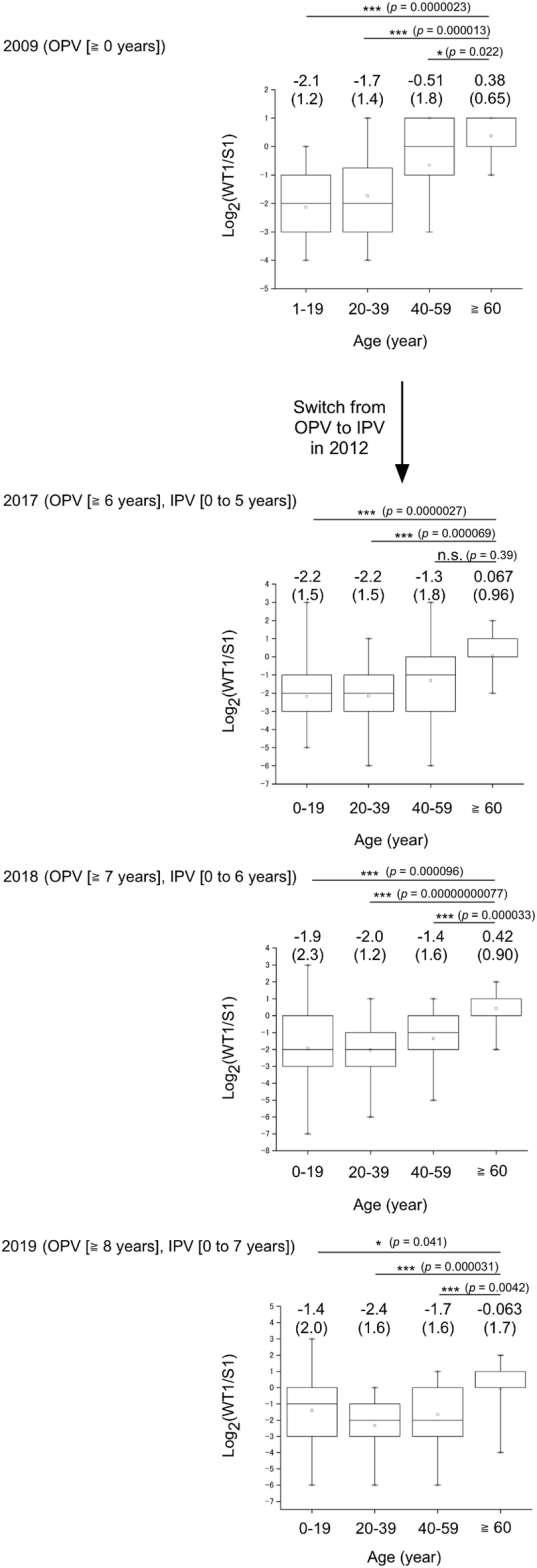
Difference of neutralizing antibody titre against type 1 PV wild-type (Mahoney strain) and that against type 1 Sabin strain among age groups. Log_2_ ratio of the neutralizing antibody titre against PV1_pv_(Mahoney) to that against PV1_pv_(Sabin 1) is shown. Each box represents 25–75 percentile with median (a line in the box), between maximal and minimal values. Values of mean and standard deviation (in parenthesis) are shown.

**Figure 5:**
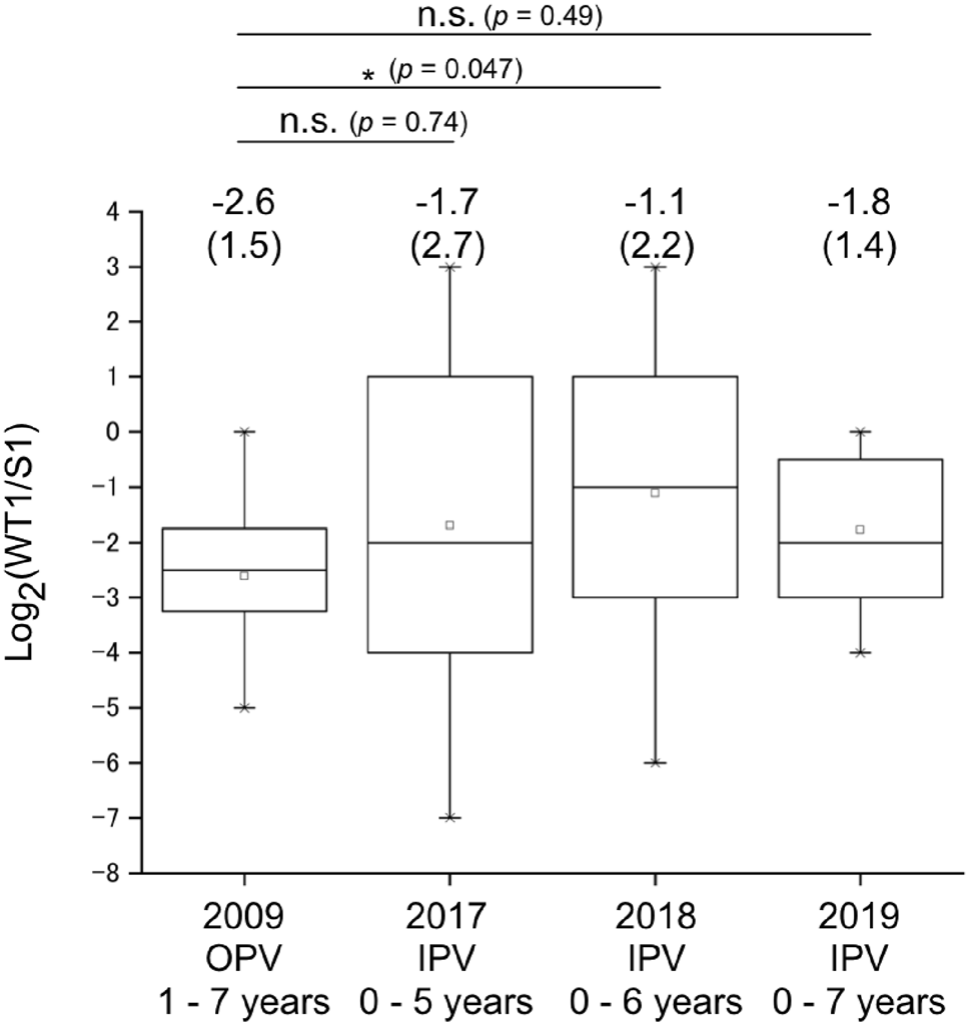
Difference of neutralizing antibody titre against type 1 PV wild-type (Mahoney strain) and that against type 1 Sabin strain between OPV and sIPV-received children. Log_2_ ratio of neutralizing antibody titre against PV1_pv_(Mahoney) to that against PV1_pv_(Sabin 1) in OPV vaccinees (serum samples collected in 2009) and sIPV vaccinees (serum samples collected in 2017, 2018, and 2019) are shown. Each box represents 25–75 percentile with median (a line in the box), between maximal and minimal values. Values of mean and standard deviation (in parenthesis) are shown.

As of 2022, only type 1 WT PV strains are circulating; type 2 and 3 WT strains are considered to be eradicated (declared by WHO in 2015 and 2019, respectively. The last cases were in India, 1999, and in Nigeria, 2012, respectively)^22,23^, while the great majority of currently circulating PV is the type 2 vaccine-derived PVs (682 cases vs. 6 cases caused by type 1 WT in 2021)^5^. Type 1 PV strains show a varied antigenicity, and the WT strains show striking difference compare to the vaccine strain, Sabin 1; neutralizing antibodies induced by the Sabin 1 strain have lower titre against the parental Mahoney strain than that to the Sabin 1 strain by approximately to four to eightfold^9,10^ and to the WT strains circulated in Africa by approximately fourfold^24^, while the WT strains circulating in Afghanistan/Pakistan had similar antigenicity to the Sabin 1 strain^25^. As a viral factor that could cause this antigenic difference, a VP3-T60K mutation in the Sabin 1 strain has been identified^26,27^. However, the human factors that could affect on the difference remains to be clarified. The national serosurveillance in Japan has been conducted by using the Sabin strains since 1984, and currently no data is available in terms of the difference in the neutralizing antibody titres against the WT and the Sabin strains. To monitor the neutralizing antibody titre against the type 1 WT strain, we performed pPNT with PV1_pv_ with capsid proteins of the type 1 wild-type (WT) Mahoney strain (PV1_pv_[Mahoney]), and compared the neutralization titre against PV1_pv_ with capsid proteins of the Sabin 1 strain (PV1_pv_[Sabin 1], Fig. 4). In the young age group, the titre against PV1_pv_(Mahoney) is approximately fourfold lower on average compared to the PV1_pv_(Sabin 1). The difference was age-dependent, and the elderly group (age of ≥ 60 years) consistently showed relatively higher titre against PV1_pv_(Mahoney) than that to PV1_pv_(Sabin 1) (approximately one to fourfold higher titre) (n = 15–16, *P* = 0.0000023–0.041). A gradual shift of the high-titre age groups (age of ≥ 40 years as of 2009) was observed between the samples collected in 2009 and samples collected in 2017, 2018, and 2019, suggesting a fixation of WT-specific immunity in the elderly group. The last poliomyelitis case by an indigenous WT strain in Japan was observed in 1980^28^. This might suggest a possibility that the elderly groups (age of ≥ 40 years as of 2009) had an asymptomatic infection by indigenous WT strains in their early childhood. Moreover, we analysed the effect of vaccine types (i.e., OPV or sIPV) in the young age group (aged 0–7 years), before and after the switch from OPV to sIPV in 2012 (Fig. 5). As a result, the vaccine types did not show significant effects on the relative titre (n = 13–45, *P* = 0.047–0.74).

In summary, we established an HTpPNT that could serve as a useful alternative to cPNT. The HTpPNT would be suitable for laboratories that perform a large-scale serosurveillance with limited manpower. Under different circumstances of country/laboratory, the HTpPNT can be implemented in the serosurveillance in combination with other proposed methods, such as cPNT with hyper-attenuated PV strains (S19), an alternative of live PV strains^29,30^. Flexibility in the implementation of the systems would help laboratories to continue the activities in the global PV eradication.

## Methods

### Cells, viruses, and human sera

RD cells (human rhabdomyosarcoma cells) and HEK293 cells (human embryonic kidney cells) were cultured as monolayers in Dulbecco’s modified Eagle medium (DMEM) supplemented with 10% fetal calf serum (FCS). Vero cells (African green monkey kidney cells) were cultured as monolayers in Eagle’s Minimum Essential Medium (EMEM) supplemented with 0.11% bovine serum albumin (BSA) (fraction V, Sigma). The RD cells were used for pPNT titration and the Vero cells were used for cPNT. PV pseudoviruses (PV_pv_s), which composed of a luciferase-encoding PV replicon based on the Mahoney strain and capsid proteins derived from PV1(Mahoney), PV1(Sabin 1), PV2(Sabin 2), or PV3(Sabin 3), were used in this study^9,11^. In house standard anti-PV serum (monkey sera, 32 U per 50 μL for each serotype of Sabin strain determined by cPNT) was used as control in pPNT. Human sera were collected from healthy volunteers (ages of 0–89) who gave informed consent by themselves, their parents or legal guardians. Experiments performed here were approved by the Committee for Ethical Regulation of the National Institute of Infectious Diseases, Japan. All experiments were performed in accordance with relevant guidelines and regulations.

### cPNT

cPNT was performed according to the standard procedure recommended by the WHO with modifications^31^ as previously described^6,9^. Briefly, a twofold dilution series of human sera was prepared with EMEM supplemented with 0.11% BSA resulting in 1/4–1/1024 dilutions. Then, 50 μL of the diluted sera or EMEM supplemented with 0.11% BSA was added to the wells of three 96-well plates (one plate for each serotype of PV with a total of three plates). Next, 50 μL of the type 1, 2, or 3 Sabin strains (100 50% cell culture infective dose (CCID_50_) ) was added to each well of the plates, and incubated at 37 °C for 3 h. After the incubation, 100 μL of Vero cell suspension in EMEM supplemented with 0.11% BSA (1.0–2.0 × 10^5^ cells) was added to each well of the plates, and the plates were incubated at 37 °C for 7 days. Neutralizing antibody titre of the serum was determined as 50% endpoints suppressing the cytopathic effect of the cells.

### HTpPNT

Basic principle of pPNT is described in previous reports^6,9^. DMEM supplemented with 1% FCS (1%FCS-DMEM) were dispensed into 384-well plates (Thermo Fischer Scientific, 264574) as follows; 45 μL/well for rows 1, 9 and 17, and 30 μL/well for other rows. Then, 15 μL of human serum samples, standard anti-PV serum, or 1% FCS-DMEM (for mock treatment) were added to the wells in rows 1, 9, and 17 (total volume of 60 μL/well) followed by 10 times manual pipetting. A twofold dilution series of the serum samples and the standard serum were prepared by using an automatic diluter/dispenser for 384-well plates by mixing eight times for the first row and then six times for other rows by the rate of 40 μL/s (EDR-384SX 12 stage work station, BIOTEC) (the final dilution of 1/4–1/512). Next, 5 μL of the diluted samples were dispensed into the wells of four 384-well plates (Greiner Bio-One, 781080) by using EDR-384SX 12 stage work station (one plate for each serotype of PV_pv_(Sabin) and PV1_pv_(Mahoney) with a total of three plates). Then, 5 μL of PV1_pv_(Mahoney), PV1_pv_(Sabin 1), PV2_pv_(Sabin 2), or PV3_pv_(Sabin 3) solution (400 IU) was dispensed into the wells of the each plate by using an automatic dispenser (WellMate, Matrix). The plates were centrifuged (200×*g*, 1 min) (5910, KUBOTA), and then incubated at 4 °C overnight. Subsequently, 20 μL of RD cell suspension (4.0 × 10^5^ cells/mL) in 5% FCS-DMEM was dispensed into each well of the plates by using an automatic dispenser (WellMate, Matrix) (total 8000 cells/well). The plates were centrifuged (200×*g*, 1 min) (5910, KUBOTA), and then incubated at 37 °C for 7 h. After the incubation, the supernatant was removed by centrifugation (1000 rpm for 1 s) using a plate centrifuge (GYRO mini, micronix), and then 5 μL of Steady-Glo solution was added to each well of the plates by using an automatic dispenser (WellMate, Matrix). Luciferase signal was measured by 2030 ARVO X luminometer (PerkinElmer). PV_pv_ infection was calculated from the percentage of luciferase signal of infected cells. The luciferase signal in mock-treated cells was taken as 100%. Neutralizing antibody titre was determined by a reciprocal number of the highest dilution of the serum that suppressed PV_pv_ infection to less than 5% (for PV1_pv_[Mahoney], PV1_pv_[Sabin 1], and PV2_pv_[Sabin 2]) or 10% (for PV3_pv_[Sabin 3]). As measures of quality controls of HTpPNT, the titre of the standard serum (less than fourfold difference from the expected titre for each type, thus > 8 and < 128), coefficient of variation of luciferase signals in mock-treated PV_pv_-infected cells (less than 30%) were used.

### Statistical analysis

Results of experiments are shown with means and standard deviations. *P values* < 0.05 by Kruskal–Wallis test and Steel–Dwass test were considered statistically significant and were indicated by asterisks (*P < 0.05; **P < 0.01; ***P < 0.001). Statistical analysis was performed by OriginPro software (regression analysis) or by R using an R commander EZR (Kruskal–Wallis test and Steel–Dwass test)^32^.

## Supplementary Information

Supplementary Information 1.

Supplementary Information 2.

Supplementary Information 3.

Supplementary Information 4.

Supplementary Information 5.

Supplementary Information 6.

Supplementary Information 7.

Supplementary Information 8.

## Data Availability

Raw data sets not included in the manuscript or the supplementary information are available from the corresponding author upon request.
